# Ambient scribe in general practice: a multi-perspective before-after longitudinal mixed-methods study

**DOI:** 10.1038/s41746-026-02454-3

**Published:** 2026-03-02

**Authors:** R. C. A. van Linschoten, C. M. van Loon, L. Joanknecht, E. W. M. A. Bischoff

**Affiliations:** 1 Department of General Practice, Erasmus MC, Rotterdam, The Netherlands; 2 Faculty of Medicine, Erasmus University Rotterdam, Rotterdam, The Netherlands; 3 Rijnmond Dokters, Rotterdam, The Netherlands

**Keywords:** Health care, Medical research

## Abstract

High workload among general practitioners (GPs) threatens clinician well-being and quality of care. Ambient scribes offer a potential solution, but evidence on their effectiveness is limited and overlooks the patient perspective. We conducted a prospective multicentre, multi-perspective, before–after longitudinal mixed-methods study on an ambient scribe in the Netherlands with 12 GPs and GPs in training without prior experience with ambient scribes. Outcomes were assessed over a two-day baseline and two-day intervention period. The primary outcome was clinical documentation time and secondary outcomes included total consultation time, documentation quantity and quality, patient and GP experiences, acceptability, and usage. Between December 2024 and July 2025, 535 patient consultations were observed. Clinical documentation time was reduced by 42.7 s per consultation (95% CI − 56.29 to −30.78; *p* < 0.0001), while total consultation time did not change. Qualitative analyses showed reduced perceived workload among GPs and, for some patients, improved communication. Potential drawbacks were inaccurate summaries, barriers for discussing sensitive information, and interference with the clinician’s reasoning process. These findings show that ambient scribing may meaningfully reduce documentation burden and support communication, but further investigation is required to understand and mitigate unintended consequences for quality and accessibility of care.

## Introduction

Increasing workload in healthcare is a major global concern, affecting clinicians across specialties and settings^1,2^. General practitioners (GPs) are particularly vulnerable to rising work demands^3^. In the Netherlands, 68% of GPs report their workload as too high, with workload increasing over the years, and nearly one in five describe their work as very or extremely stressful^3^. This chronic strain has resulted in significant staffing challenges: over half of Dutch GP practices struggle to recruit locum GPs or support staff, and 60% have stopped accepting new patients^4–6^. High workload negatively affects both provider well-being and the quality of care delivered, impacting job satisfaction, patient-provider communication, healthcare costs, care quality, and patient safety^7–9^.

A major contributor to workload is the administrative burden^3^. GPs in the Netherlands spend only 60% of their time on direct patient care, with the remaining 40% devoted to indirect or non-patient-related activities, such as documentation and administrative tasks^6^. Increasing time spent on clinical documentation is linked to higher stress levels and reduced time for patient interaction, key drivers of physician dissatisfaction and burnout^4,10,11^.

Reducing the time GPs spend on documentation has the potential to improve physician well-being and patient care. Recently, ambient scribes have emerged as a promising tool. These systems use large language models (LLMs) to passively record and transcribe medical consultations in real-time, generating clinical documentation with minimal physician input. This would allow physicians to focus on patient interaction while the system handles documentation in the background.

Research on ambient scribes has shown some promising benefits for clinical practice. Retrospective before-and-after studies using electronic health record (EHR) log data suggest that ambient scribes may reduce documentation time^12–16^. However, these studies are not controlled and may be subject to bias. The only study using the gold standard of continuous external observation found no significant change in documentation time when using an ambient scribe^17,18^. Similarly, a different study evaluating productivity also reported no change when an ambient scribe was introduced^19^.

Most evaluations of ambient scribes have reported reduced perceived workload and cognitive burden among clinicians^12,14–16,20–24^, though some studies found no effect^18,25^. Evidence regarding communication and patient experience is mixed: while some studies report improved communication^14,21–24^, others found no change^12,19^. Importantly, almost all research on communication has focused on the clinician perspective. Only one study included the patient experience data via a survey, and none employed qualitative methods to explore the patient experience in depth.

Despite growing interest in ambient scribes, existing evidence is limited in both scope and methodological quality. Most studies use retrospective designs and rely on indirect measures, with little attention to the patient perspective. Consequently, the impact of ambient scribes on efficiency and care quality remains insufficiently understood. To address these gaps, we conducted a study to comprehensively evaluate the use of ambient scribes in clinical practice from both the provider and patient perspectives.

## Results

### Baseline characteristics

**Fig. 1:**
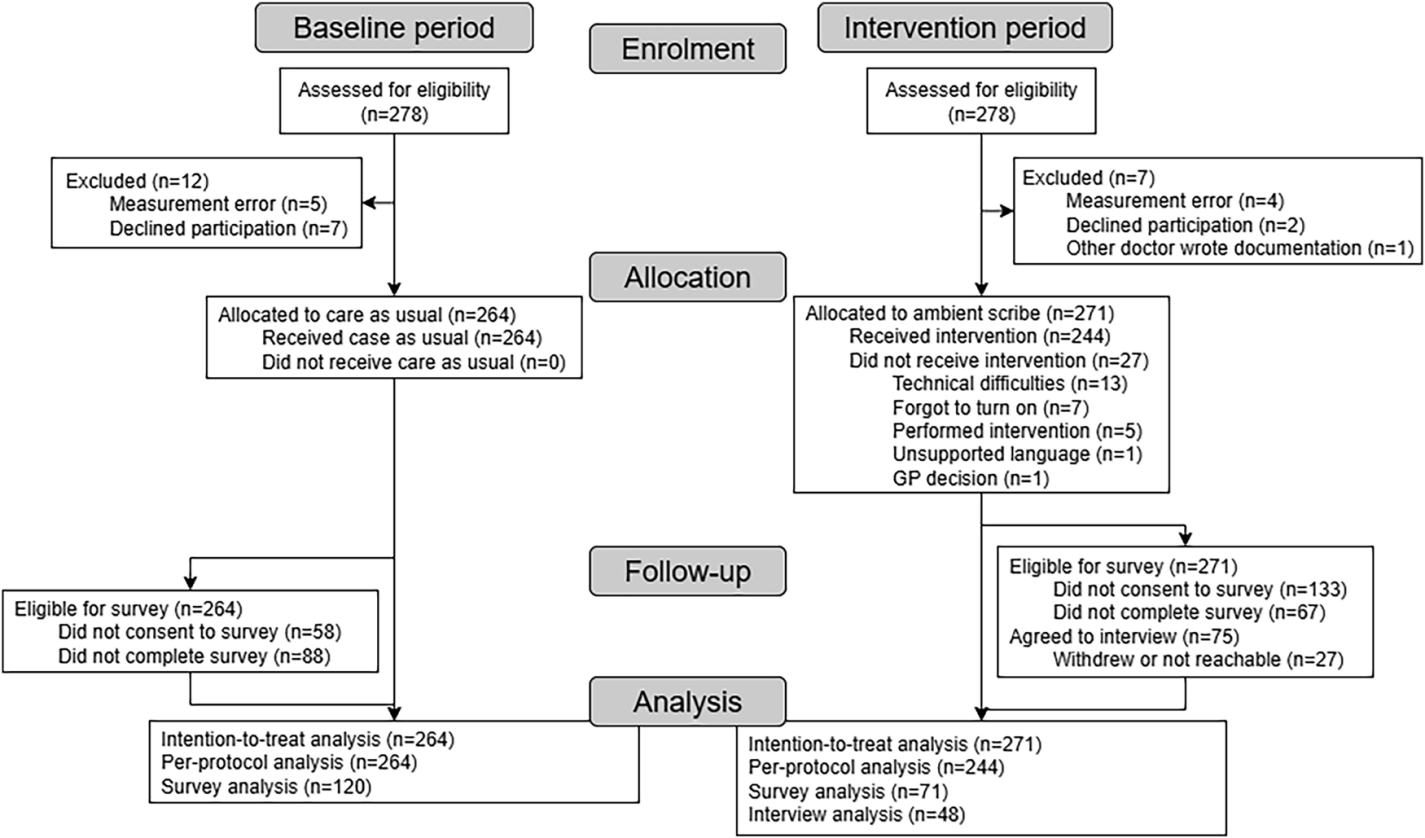
Flowchart of patient inclusion. The diagram illustrates the flow of GP consultations through the baseline and intervention periods in this prospective before–after study. During each period, consultations were assessed for eligibility, with exclusions due to measurement errors, declined participation, or protocol deviations. Eligible consultations were allocated to care as usual or the ambient scribe intervention, with reasons for non-adherence to the intervention detailed. Follow-up shows eligibility and participation in surveys and interviews, including non-consent and attrition. The analysis stage summarises the number of consultations included in intention-to-treat, per-protocol, survey, and interview analyses for both periods.

Between December 9, 2024, and July 2, 2025, twelve GPs participated in the study, contributing a total of 535 observed consultations (Table 1; see also Fig. 1 and Supplementary Table 2). Most consultations addressed a single complaint, though some involved multiple issues. In total, 333 complaints were recorded in 264 consultations during the baseline period and 339 complaints in 271 consultations during the intervention period.

**Table 1:** Baseline characteristics of GPs

Characteristic^a^	*N* = 12
GP characteristics	
Age (years)	39 (37, 49)
Female	8 (67%)
Experience as a GP (years)	8 (5, 15)
Digital skills	
General digital skills	9.30 (8.20, 9.90)
EHR skills	7.60 (5.15, 8.40)
Skills in using various programs	8.60 (6.85, 9.45)
Skills for safe internet use	7.80 (7.35, 8.75)
UTAUT	
Outcome expectation	4.00 (3.25, 4.75)
Effort expectation	4.00 (3.75, 4.75)
Social influence	3.38 (2.88, 3.63)
Facilitating conditions	3.50 (3.25, 4.00)
Intention to use	4.00 (3.33, 4.33)
Practice characteristics	
Type	
Duo practice	3 (25%)
Group practice	9 (75%)
List size	5791 (4733, 6585)
Location	
Rural	3 (25%)
Semi-rural	2 (17%)
Urban	7 (58%)
Patients from a deprived area	2% (2%, 4%)
Direct link between tool and EHR	2 (17%)

*EHR* electronic health record, *GP* general practitioner, *UTAUT* Unified Theory of Acceptance and Use of Technology.

^a^Median (Q1, Q3); *n* (%).

The majority of consultations were scheduled for 15 min, 91% during the baseline period and 89% during the intervention period. General and unspecified complaints were more frequent in the baseline period (absolute percentage difference: +4.6%), along with ear issues (+2.5%). In contrast, complaints related to the nervous system (+3.2%) and skin (+2%) were more commonly discussed during the intervention period (Supplementary Table 3). Missing data were minimal for most variables, except for survey responses (see Table 1 and Supplementary Tables 2 and 3).

### Primary outcome

During the intervention period, the mean time spent on documentation per consultation was 42.7 s shorter than in the baseline period (95% CI: −56.29 to −30.78; *p* < 0.0001).

### Quantitative outcomes

The total consultation time showed no significant difference (−61.4 s, 95% CI: –131.91 to 0.96; *p* = 0.069). For distribution of time variables between the baseline and intervention period, see Supplementary Table 4. There was an increase in the length of the subjective, assessment and plan parts of the clinical note in the intervention group, but not in the objective part. In the intervention period, there were more sign and plan variables noted in the documentation, but less symptom and measurement variables, and no significant difference was found in the number of context and diagnosis variables. No differences were found in patient experience as measured with the patient experience questionnaire between the intervention and baseline period. See Table 2 for all estimates, confidence intervals, and *p*-values.

**Table 2:** Parameter estimates, 95% CIs and p-values of the main analyses

Name	Estimate	95% CI	*p*-value
Time outcomes^a^			
Documentation time	−42.72	[−56.29; −30.78]	<0.0001
Consultation time	−61.38	[−131.91; 0.96]	0.069
Note length^b^			
Subjective	1.15	[1.04; 1.27]	0.005
Objective	0.87	[0.73; 1.04]	0.131
Assessment	1.19	[1.01; 1.4]	0.036
Plan	1.57	[1.41; 1.75]	<0.0001
Number of variables in note^b^			
Signs	1.12	[1.01; 1.22]	0.036
Context	1.03	[0.89; 1.18]	0.621
Symptoms	0.80	[0.69; 0.92]	0.004
Diagnosis	0.97	[0.84; 1.14]	0.713
Plan	1.18	[1.08; 1.29]	0.0004
Patient experience^a^			
Outcome of the consultation	0.10	[−0.14; 0.34]	0.427
Communication experiences	0.11	[−0.09; 0.33]	0.286
Absence of communication barriers	−0.03	[−0.22; 0.17]	0.773
Staff relationship	0.03	[−0.16; 0.23]	0.770
Emotions after consultation	0.08	[−0.28; 0.45]	0.682

*CI* confidence interval.

^a^Difference on an additive scale.

^b^Difference on a multiplicative scale.

The sensitivity analyses adjusted for total documentation time showed the same results (Supplementary Table 5). The only difference between the per-protocol analysis and intention-to-treat analyses was that in the per-protocol analysis the number of context variables in the note was higher in the intervention period than in the baseline period (Supplementary Table 6). The post-hoc sensitivity analysis adjusted for ICPC code agreed with the main analyses (Supplementary Table 7). In the post-hoc analysis for EHR integration, the ambient scribe reduced total documentation time by 41.70 s (95% CI: −57.58 to −28.41; *p* < 0.0001), while integration of the ambient scribe in the EHR did not lead to a further reduction of total documentation time (−4.40 s; 95% CI: −33.03 to 26.45; *p* = 0.769). The post-hoc analysis of learning effects showed a reduction of total documentation time due to the ambient scribe of 29.38 s (95% CI: −50.74 to 9.02; *p* = 0.004), and a reduction for each subsequent consultation of 1.06 s (95% CI −2.24 to 0.08, *p* = 0.089).

### Qualitative outcomes

Nearly all patients in the intervention group were invited to participate in interviews, with a few exceptions due to logistical issues, emergency consultations, or a language barrier. Of the 75 patients who initially agreed, 48 were interviewed (Fig. 1). Participant demographics are presented in Supplementary Table 8. No new themes emerged in the final interviews, suggesting that data saturation was reached. Thematic analysis identified six overarching themes: process of using the tool, communication, clinical documentation, the consultation, safety of the tool, and workload (Table 3).

**Table 3:** Themes, subthemes and definitions from the thematic analysis of interviews with patients and GPs

Main theme	Sub-theme	Definition
Process		The integration of the ambient scribe within existing workflows
	Activating the tool	The process of activating the ambient scribe before the consultation.
	Direct link	The existence of a direct link between the ambient scribe and the EHR.
	Noticing the tool	Noticing the ambient scribe’s presence during the consultation.
Communication		The effect of the ambient scribe on the conversation between patients and the GP
	Interaction	The quality and nature of interaction between the patient and the GP.
	Sensitive topics	The ability to discuss personal or difficult subjects without barriers.
Clinical documentation		The summary made by the ambient scribe
Consultation		The effect of the ambient scribe on the consultation
	Quality	The overall quality of the consultation.
	Explanation of plan	The amount of time spent explaining the diagnosis or treatment plan
	Distraction of GP	Whether the ambient scribe distracts the GP
	Distraction of patient	Whether the ambient scribe distracts the patient
	Time for asking questions	The amount of time available for patients to ask questions
Safety of the tool		Concerns regarding data privacy, AI reliability and trust in AI applications in healthcare.
	Trust privacy tool	Patients’ and GPs’ privacy concerns
	Trust in AI in general	General confidence in artificial intelligence and its applications in healthcare.
Workload		Refers to how the ambient scribe affects the GPs job and workload
	Workload impact	How the ambient scribe affects workload
	Job satisfaction	How the ambient scribe affects job satisfaction.
	Time savings	Whether the ambient scribe saves time

*AI* artificial intelligence, *EHR* electronic health record, *GP* general practitioner.

#### Process of using the tool

Several GPs reported that, because the tool was not directly integrated into their EHR, they occasionally forgot to activate the tool before the consultation started, particularly in urgent or complex cases. The need to manually copy and paste the generated summaries into the EHR was generally perceived as a minor inconvenience, requiring little additional time. However, GPs with lower levels of digital proficiency found this more burdensome. GPs using the integrated version also reported limitations. These were the inability to automatically transfer measurement data to the vital signs section and to produce separate summaries for consultations with multiple complaints.

Most patients did not notice the presence of the microphone or the use of the tool during consultations. Similarly, most GPs reported that patients were generally unaware of the tool and microphone, with almost none making any remarks about its presence.

#### Communication

Some GPs suggested that less typing during consultations could enhance patient interaction, reduce screen time, and foster a more continuous connection with the patient through increased eye contact. One GP also noted that using the tool might help ensure that subtle patient cues or interactions are less likely to be missed. However, almost all GPs reported that they barely typed during consultations and thus perceived no difference in their consultation dynamics or patient interaction.

Most patients reported no difference in interaction compared with previous consultations, noting that GPs maintained attention, eye contact, and communication as usual. This was largely attributed to the fact that GPs already devoted sufficient attention and time to their patients during consultations. However, some patients felt they received more attention from their GP, with a few noticing increased eye contact during the consultation. They described this as contributing to a better conversation, making it easier to reach the core of their concerns and fostering a stronger sense of connection and trust in the GP.

Most GPs and patients noted that the tool could potentially pose a barrier for patients, particularly during consultations involving sensitive topics such as domestic violence, relationship problems, addiction, STDs or mental health issues. A few patients mentioned that they already found it difficult to talk about psychological problems and that the presence of the tool might slightly increase this barrier. GPs emphasized that they could easily deactivate the tool when a patient felt uncomfortable. When patients were directly asked whether they personally felt inhibited in discussing such topics while the tool was active, most reported that they did not experience any barriers. They indicated that they would feel comfortable discussing their complaints even if the tool was used in future consultations. Although several GPs and patients expected that older adults or people from different cultural backgrounds might experience greater discomfort, we observed no differences in the interviews between age groups, ethnic backgrounds, or education levels.

#### Clinical documentation

Most GPs acknowledged that the AI-generated summaries were not always completely accurate, requiring them to review and adjust nearly every summary to ensure there were no errors. Several GPs reported that essential information or risk factors were occasionally missing, and one GP continued to make personal notes because he could not rely on the system. Almost all GPs mentioned that the physical exam was not correctly summarised by the ambient scribe tool, requiring them to manually type this section. The overall quality of the summaries tended to be lower for follow-up consultations, consultations involving more persons, phone calls, patients who switched between languages, or cases in which multiple complaints were discussed. However, other GPs noted that the tool was able to distinguish multiple complaints effectively in some situations. Overall, GPs noted that it was convenient to have most of the summary already written down, and they only needed to make the necessary adjustments. Some GPs found the AI-generated summaries particularly valuable for longer or more complex consultations, such as those with persistent somatic symptoms or psychiatric complaints, whereas others considered them more accurate and useful for shorter and simpler consultations. Nonetheless, many GPs found the ability to adjust the AI-generated summary using different templates very useful, for example by modifying the reason for encounter or refining the medical terminology.

Some patients suggested that using an ambient scribe tool could improve clinical documentation by making it more detailed, objective, and less influenced by the GP’s interpretation. Other patients expressed concerns that the summary might not capture the full essence of the conversation. Since the tool does not understand non-verbal interaction, it may miss nuances such as sarcasm or facial expressions. Nevertheless, other patients pointed out that GPs review and edit the notes before entering them in the EHR and therefore believed this would not affect the overall quality of the documentation.

#### Consultation

Most patients thought the tool could give GPs more time to explain the diagnosis or treatment but did not believe it enabled them to ask more questions. When specifically asked, many reported no difference compared with previous consultations, as they felt there was already sufficient time for explanation and questions. Similarly, GPs stated that they always tried to take enough time to explain the diagnosis or treatment plan, with no noticeable difference when using the ambient scribe. A few patients believed that any time savings mainly occurred outside direct patient contact, allowing GPs to handle more administrative tasks, see additional patients, or address more complaints within the same consultation.

Almost all GPs reported that the tool did not cause any distraction during the consultation, with only a few exceptions, such as when the wrong language was selected. All patients confirmed that they did not notice the GP being distracted by the tool. Both GPs and patients generally agreed that the tool did not distract the patients, as most were unaware of its use.

Some GPs also mentioned that using the tool could enhance the overall quality of the consultation. They explained that it helped them think in a more structured way, and the generated summary served as both a memory aid and as a means to verify with patients whether the information had been recorded correctly. It also ensured that consultations were documented immediately, preventing delays in completing notes. However, one GP noted that summarising was part of their clinical reasoning process and that this could be lost when using the tool. Another GP felt that the quality of the consultation decreased, as the summaries became less accurate when the conversation deviated from the main topic or included small talk, making the GP less likely to engage in such interactions.

#### Safety/privacy of the tool

Some patients and GPs expressed concerns about the safety of the tool, particularly regarding whether the transcripts used to generate the summaries were deleted immediately, where they were stored, and how securely they were protected. They were specifically worried about the potential risk of data breaches. However, most patients and GPs were not particularly worried about data security, with many patients expressing trust in their GP and confidence that confidential information is well protected within the Dutch healthcare system.

#### Workload

Some GPs mentioned that the tool helped reduce their workload, noting that it was less demanding and required less cognitive effort to adjust the AI-generated summary than to type it themselves. A few GPs added that if the summaries would be more accurate, the tool could further improve efficiency. Most patients thought that the tool could reduce the workload for GPs. Some GPs did experience an increase in work satisfaction, noting that not having to do clinical documentation made consultations easier. Other GPs appreciated trying out new technologies, but the tool did not contribute to an increase in work satisfaction.

Most GPs experienced spending less time on clinical documentation and were running less behind on their consultations. One GP mentioned that the additional time gained was used to address more complaints within a single consultation, while others used it for practice related tasks. However, one GP found that the tool occasionally increased workload, particularly when seeing patients briefly between scheduled appointments or when additional time was required to reread and correct the summary. A few GPs also noted that, as they normally typed while patients were getting undressed for the physical exam, they now spent that time waiting, which felt less efficient. The tool was further described as inconvenient when making a referral, as the summary had to be completed while the treatment plan was often still being discussed. Some GPs mentioned they usually did not explain the tool to patients, as doing so would also take additional time. Almost all patients could imagine that the use of the ambient scribe tool for clinical documentation would save time, though some mentioned that correcting the summary could be time-consuming.

## Discussion

In this prospective, multicentre, longitudinal before–after mixed-methods study, we found that the use of an ambient scribe reduced documentation time in general practice. However, it did not affect total consultation time or patient experience. During the intervention period, documentation length increased, with more signs and plans recorded but fewer symptoms. The tool was applied in most consultations, except when technical issues arose, the GP forgot to activate it, or an intervention was performed. While the ambient scribe reduced GP workload, neither patients nor GPs perceived improvements in communication. The generated summaries were not always fully accurate, often missed non-verbal cues, and required GP review in all cases.

The research on ambient scribes has rapidly increased with the widespread use of LLMs^12–16,18–24^. However, most studies rely on aggregated retrospective data or surveys to measure time outcomes and do not adjust for differences between treatment and control periods. In contrast, we used continuous external observation, the gold standard for measuring time outcomes, and demonstrated that an ambient scribe reduced documentation time by 42.7 s per consultation. This finding remained robust in both per-protocol and (post-hoc) sensitivity analyses, which adjusted for total documentation time and reason for consultation^17^. Our study, using gold standard methodology and adjusted analyses, confirms the results of prior studies reporting a reduction in documentation time^12–16,23^. In our post-hoc analysis for learning effects, we found a non-significant reduction of 1.06 s for each subsequent consultation. Future studies should assess whether learning periods persist over longer durations and whether the effect of the ambient scribe can be further improved over time.

No difference in total consultation time was observed in the intention-to-treat analysis, with the effect estimate moving closer to zero in both the per-protocol and the post-hoc sensitivity analysis adjusted for reason for consultation. A possible explanation is that reductions in documentation time were offset by GPs spending more time on other parts of the consultation, for example, by expanding the medical history or taking more time to explain the diagnosis and treatment. We also observed that GPs used a period of downtime during consultations to type the summary, which may have diminished the ambient scribe’s effect on reducing total consultation time. A different explanation is that, while reduced documentation time may shorten the overall consultation, the high variance in total consultation time could have limited the statistical power to detect such an effect. Future studies should base their sample size calculations on total consultation time and examine how the use of an ambient scribe influences different components of the consultation to better understand its impact on time outcomes. Integration of the ambient scribe into the EHR did not reduce documentation time significantly as shown by our post-hoc analysis. However, it may further reduce cognitive burden as GPs saw copying and pasting as a minor inconvenience.

In interviews, GPs mostly described the reduction in documentation time as leading to lower workload and improved work satisfaction, helping them feel less tired and less behind on consultations. These observations are consistent with previous research showing that ambient scribes can reduce burnout, cognitive burden, and task load^12,14–16,20,21,23,24^. Considering our quantitative and qualitative findings together with prior research, ambient scribes appear most effective in reducing workload and improving job satisfaction. Because the observed time reduction of 42.7 s is relatively small, and most GPs did not experience a net time savings, ambient scribes seem less effective at shortening consultation length or enabling providers to see more patients per day. Overall, in this study, ambient scribes appear to be more effective in reducing cognitive burden than in improving operational efficiency.

Our study indicates that the use of an ambient scribe can have both positive and negative effects on patient–doctor communication. On one hand, some patients reported that the tool enhanced their sense of connection and trust, citing increased eye contact and better conversation. However, no differences were observed in the patient-experience questionnaires, suggesting that if the ambient scribe enhances communication, the effect likely applies only to a subset of patients. In contrast, most GPs did not perceive an improvement, noting that they already typed minimally during conversations, typically using idle moments for documentation. Previous studies, mostly from the healthcare provider’s perspective, have shown mixed evidence on this topic, with some reporting improved communication and others finding no effect^12,14,19,20,24^. One study from the patient perspective found that providers spend less time on the computer, were more focused on the patient and that they had a more personable conversation^22^. Future research should use more objective measures to assess whether ambient scribes can mitigate the impact of the EHR on communication^26^.

Conversely, some patients felt that the presence of an ambient scribe could create a barrier when discussing sensitive issues. Although most participants did not feel inhibited, there may be a small group of patients that are impacted. As these are often topics that patients already found hard to discuss, such as domestic violence, addiction or mental health, the use of an ambient scribe could further reduce accessibility for an already vulnerable population.

When exploring which patients might be impacted, we found no differences across age, background, or education. However, summary quality was lower for patients who switched between languages during the consultation, suggesting a potential accessibility concern for patients with migratory backgrounds. Prior research indicates that digital tools may exacerbate health disparities, underscoring the need for further research to ensure that the implementation of ambient scribes does not compromise accessibility of care for these vulnerable subgroups^27^.

When comparing notes generated by the ambient scribe with those written by the GP, we observed that the notes of the ambient scribe were generally longer across most sections. However, they contained more substantive information only in the signs and plan sections. This raises the question whether the increased length contains beneficial information or is just verbosity contributing to so-called “note bloat”^28^. At the same time, fewer symptom variables were recorded. Interviews revealed that this occurred because the ambient scribe often failed to correctly summarise the physical examination, requiring the GP to add this information manually. This was attributable to the clinician not verbalizing these results aloud in sufficient detail. Yet, even with the manual additions of the GP, fewer symptom variables were ultimately documented.

A possible explanation is that, because the scribe already produces summaries for the other sections, GPs may invest less effort in completing the physical examination notes than they would if they were writing the entire record themselves. This may create an overreliance on the scribe, with insufficient correction by the GP, reducing the quality of documentation for this part of the consultation. Manual adjustment of the summaries by a clinician is thus not necessarily an adequate safeguard for documentation quality.

Future use of ambient scribes may thus lead to degraded documentation quality because of missing information on signs and measurements, which warrants further study. Possible solutions include encouraging GPs to verbalize the physical examination, simultaneously informing the patient of their findings, or enabling ambient scribes to use standard templates that are dynamically adjusted based on information captured during the visit.

The problem may be compounded by the fact that ambient scribes occasionally hallucinate, requiring GPs to carefully review and adjust each summary, an issue also reported in earlier studies^14,21,24,25^. Patients likewise expressed concerns that the scribe might miss sarcasm, non-verbal cues, or unspoken reflections from the GP. Future research should therefore assess documentation quality not only by comparing notes with consultation transcripts, but also by examining verbosity, safety, practical value for healthcare providers who use them in follow-up care and value for patients when reading back their own records.

Ambient scribes may have mixed effects on the clinical reasoning process. Some GPs perceived an improvement, noting that the scribe functioned as a memory aid and allowed them to verify information directly with the patient. However, one GP expressed concern that delegating note-taking might remove an important moment of reflection that typically occurs when clinicians summarise the consultation themselves. Given that there is an important role of summarisation in the clinical reasoning process, future research should explore this potential unintended consequence^29^.

The main strength of our study is that, to our knowledge, it is the first prospective mixed-methods investigation of ambient scribing to employ gold-standard methodology for time measurements alongside a multi-perspective qualitative and quantitative design. This approach enables triangulation of findings and provides a richer understanding of ambient scribe use from both provider and patient perspectives, an aspect not addressed in prior LLM-based trials. Although our study focused on general practitioners, the results are likely transferable to other outpatient settings, including medical specialty care, as the findings are consistent with previous research on ambient scribing in other contexts^12–24^. Importantly, our study extends the existing evidence base by delivering more precise estimates of time-related outcomes and by identifying potential adverse effects not previously documented, including impact on clinical reasoning, reduced detail in the documentation of symptoms and measurements, and patient perceptions of the technology.

Some limitations should be acknowledged. First, participation was limited to GPs who were willing to adopt ambient scribing. These GPs may have held more favourable attitudes toward ambient scribes, potentially influencing both their experiences and overall usage rates. Second, most participating practices served relatively few patients from deprived areas, and the majority of interviewed patients had higher education levels and a Western background. Individuals with lower SES and diverse migration backgrounds were underrepresented. Prior research indicates that patients from lower SES groups may show greater distrust toward AI in healthcare, often linked to concerns about data privacy or limited familiarity with the technology^30^. This underrepresentation may reduce generalizability of our findings. Third, the GPs in our sample scored highly on both digital skills and UTAUT questionnaires and were relatively young. Our results may not fully capture the perspectives of older GPs or those less experienced with digital tools. Future research should make a concerted effort to include these underrepresented groups and conduct subgroup analyses to evaluate the effectiveness and acceptability of ambient scribing across different populations. This is particularly important given the risk that digital innovations may inadvertently widen existing health disparities. Fourth, our study included only GPs and did not involve other types of healthcare providers. Future research should recruit participants from a broader range of healthcare sectors to generate findings that are more generalizable across the healthcare system. Including diverse provider groups would also enable cross-sector comparisons, helping to assess the usability of ambient scribes in a wider variety of settings. Fifth, the two-day observation period for both the baseline and intervention phases may be too short to fully capture the natural variation in clinical workflow. Sixth, the summaries produced by both GPs and the ambient scribe were not evaluated against a reference standard. Although our measures of documentation quantity and quality revealed differences between the baseline and intervention periods, more comprehensive assessment tools could have provided deeper insight into the technical reliability of the ambient scribe. Seventh, the lack of blinding for both participants and the observer may have introduced bias away from the null. However, given the nature of the study, blinding was not feasible for either group.To increase the impact of ambient scribes in healthcare, developers should prioritize the following areas. First, integration with the EHR to minimize the time spent copying and pasting summaries. Second, improving the accuracy and quality of the generated summaries, particularly regarding symptoms and measurements, to further reduce the time and cognitive load clinicians spend on summarisation and to prevent less detailed documentation of these variables. Third, adding advanced functionalities based on the content of the summary, such as drafting a referral or ordering diagnostic tests, to lessen the administrative burden on healthcare providers.

Concluding, ambient scribing in general practice reduces documentation time and workload, improves job satisfaction, and potentially patient–provider communication. However, it also results in less detailed documentation of clinical signs in the EHR, does not increase patient throughput, could reduce accessibility for patients with sensitive issues, and it may interfere with aspects of the clinical reasoning process.

## Methods

### Study design

This prospective multicentre multi-perspective longitudinal before-after mixed-methods study examined the effect of an ambient scribe in general practice. This study was conducted in general practices across the Netherlands between December 2024 and July 2025. The medical ethical review committee of the Erasmus MC deemed the study to not be subject to the Medical Research Involving Human Subjects Act and provided a waiver (MEC-2024-0286). All study participants provided informed consent prior to inclusion in the study. This trial was registered at ClinicalTrials.gov, NCT06691724.

### Participants

The study included two participant groups: GPs or GPs in training who were interested in implementing the ambient scribe and patients who had consultations with these GPs. GPs were recruited through regional care groups in the Netherlands and the professional networks of the authors. GPs were purposively selected to ensure diversity in both patient populations (e.g., age, migration background, gender) and GP demographics, with the goal of obtaining a representative and generalizable sample. GPs with prior experience using AI-based transcription and reporting tools during consultations were excluded from participation.

### Intervention

The intervention under study was the Juvoly QuickConsult transcription tool (www.juvoly.nl). This software records and transcribes consultations using a microphone and an LLM. Hereafter, an LLM generates a structured summary of the consultation based on the Subjective, Objective, Assessment, and Plan (SOAP) framework. SOAP is the standard format for clinical documentation in general practice. The tool processes each conversation in the cloud, converting speech to text in real time without storing audio, transcripts, or clinical notes, and all data remains under the control and ownership of the healthcare provider. In some EHRs, Juvoly QuickConsult is directly integrated. When integration was unavailable, GPs used the website from Juvoly QuickConsult and manually copied and pasted the generated summary into their EHR.

### Timing

The study was conducted across three consecutive phases: a baseline period, an implementation period, and an intervention period, each lasting two weeks. During the baseline period, GPs were observed over two full days or three half-days of consultations without using the ambient scribe. During the implementation period, GPs received a brief explanation and demonstration of the tool, followed by time to practice using it. During the intervention period, GPs were again observed for two full days or three half-days while using the ambient scribe. To reduce natural variation between the baseline and intervention periods, GPs were observed on the same weekdays in both phases.

### Outcomes

The primary outcome was the time spent on clinical documentation in seconds, which included time waiting for the tool to generate summaries, time spent copying and pasting the summaries into the EHR if applicable, and time spent on editing and reviewing the generated summaries. Secondary outcomes included total time per consultation in seconds, the quantity and quality of documentation, GP and patient experiences, the acceptability of the tool, and the tool usage rate. For training and validity of outcome assessors, see the Supplementary Methods. Investigators, patients, and providers were not blinded to intervention allocation as this was not feasible.

During both the baseline and intervention period, time outcomes were assessed through direct observation of GP consultations. An external observer continuously recorded the time spent on different tasks using specialized software (www.timecat.org). Tasks were categorized as either communication-related or hands-on; definitions for each task type are provided in Supplementary Table 1. Multitasking was calculated by measuring the overlap between communication and hands-on tasks.

Documentation quantity was reported as the number of words used in each SOAP-item. Documentation quality was assessed by a single researcher by counting the number of relevant clinical variables in the note, which were categorized by type: signs and related variables (e.g. site, onset, character, time course), contextual factors (e.g. self-care, risk behaviour, family history), symptoms and measurements (e.g. physical examination, pulse, temperature), (differential) diagnosis or plan variables (e.g. prescriptions, referrals, safety net).

Patient experiences were assessed during both the baseline and intervention period using the Patient Experience Questionnaire (PEQ)^31^. This survey asked patients to evaluate their experiences during the consultation with the GP across different categories: medical content, communication, general experiences, and their emotions immediately after the consultation. Scores for medical content, communication, and general experiences ranged from 1 (indicating a negative outcome) to 5 (indicating a positive outcome), while emotions were rated on a scale from 1 (negative emotion) to 7 (positive emotion).

To gain deeper insight into the experiences of both patients and GPs with the ambient scribe and its impact on the consultation, semi-structured interviews were conducted during the intervention period. These interviews were guided by a predefined topic list (see Supplementary Methods). Interviews were conducted face-to-face with all participating GPs. Patients were approached after consultations and asked whether they would be willing to participate in an interview. Those who agreed received an information letter about the study, and were invited for an interview. Patient interviews were conducted face-to-face, via Microsoft Teams, or by phone, preferably within the same week as the consultation. Each interview lasted around 10–20 min and was audio-recorded and transcribed using Microsoft Teams. Transcripts were not returned to patients or GPs for comments or corrections.

The acceptability and usage of the tool by GPs were assessed during the baseline, implementation, and intervention period using the Unified Theory of Acceptance and Use of Technology (UTAUT) questionnaire^32^. The questionnaire covers outcome expectation, effort expectation, social influence, facilitating conditions, and intention to use the tool. Scores ranged from 1 (negative outcome) to 5 (positive outcome).

For each GP included in the study, the following baseline characteristics were collected: gender, age, years of experience as a GP, type of practice, patient population size, percentage of patients from deprived areas, and whether the ambient scribe was directly integrated into the EHR. Digital skills (categorized in general digital skills, EHR skills, skills for safe internet use, and competencies using various programs and applications) were assessed using the Dutch digital skill questionnaire for general practice (www.digivaardigindezorg.nl/huisartsen/home/zelftest/). The geographic area of the practice was defined according to the criteria of the Dutch Central Bureau of Statistics (CBS, www.cbs.nl/nl-nl/onze-diensten/methoden/begrippen/stedelijk-gebied).

For each patient observed in the study, age and gender were collected. We also collected the International Classification of Primary Care (ICPC) codes linked to the consultation from the EHR^33^. For each patient interviewed, we also collected gender, educational level, migration background, language proficiency, and presence of long-term health conditions.

### Sample size

Sample size calculations were based on simulations that explored various assumptions to assess their impact on the required sample size. The assumptions included:

- Average consultation duration of 10 or 15 min, with a standard deviation (SD) of 5 min^34,35^.
- Average documentation time equal to 35% of the total consultation time (SD 5%), as well as a more conservative estimate of 17.5% (SD = 2.5%)^36,37^.
- A normally distributed random effect at the GP level to account for variation in documentation time, with an SD of 1 or 2 min.
- A minimum documentation time of 10 s per consultation.
- A reduction in documentation time of either 25% or 50% due to the use of the tool.

Even under the most conservative assumptions: a documentation time of 1.75 min, a GP-level random effect of 2 and an effect size of 0.75, 10 consultations each for 3 GPs would provide a statistical power of 99%.

To enhance the generalizability of our findings, we aimed to include at least ten GPs from at least five different practices. For each GP, we observed consultations over two full days or three half-days, yielding approximately 40 consultations per GP and an expected total of 400 observations per study period. The sample size for interviews varied: we interviewed all participating GPs and all patients who expressed willingness to be interviewed.

### Statistical analysis

We conducted an intention-to-treat analysis comparing consultations during the intervention period with those from the baseline period. All data were analysed as observed. The impact of the ambient scribe on clinical documentation time and total consultation time was assessed using linear mixed models. These models accounted for clustering by including a random effect for each participating GP and a fixed effect for planned consultation duration.

To evaluate documentation quantity and quality, we used negative binomial generalized linear mixed models with a log link function, appropriate for the distribution of the outcome variables. These models also included a random effect for participating GPs and a fixed effect for planned consultation time. Patient experience questionnaires were analysed using linear mixed models with random effects for participating GPs. All random effects were assumed to follow a normal distribution. Tool usage rates and acceptability were summarised using descriptive statistics.

Three sensitivity analyses were performed. First, a per-protocol analysis was conducted for time, documentation and patient experience outcomes. In the per-protocol analysis, we included consultations from the baseline period only when the tool was not used, and from the intervention period only when the tool was used. Second, we included an additional adjustment for total non-documentation time when analysing documentation time and documentation-related outcomes. This variable was considered a potential confounder for two reasons. Within the fixed 10- or 15 min consultation window, reduced patient-facing time may increase the time available for documentation. Moreover, more extensive in-consultation discussions may lead to increased documentation. Third, because there were differences in the reasons for consultations between the baseline and intervention period, a post-hoc sensitivity analysis was conducted for time outcomes, which we adjusted for ICPC code. Fourth, to assess the effect of integration of the ambient scribe in the EHR on documentation we performed a post-hoc analysis in which we added a variable for integration of the ambient scribe. Fifth, to assess potential learning effects, we conducted an additional post-hoc analysis in which we included an interaction term indicating the ordinal number of the consultation performed with the ambient scribe.

All quantitative analyses were conducted using R (version 4.4.3) within the R-studio environment (version 2025.05.0). Mixed models were fitted using lme4, lmerTest was used for *p*-values and 95% bootstrap confidence intervals (CI), and model diagnostics were assessed using the DHARMa package^38–40^.

### Qualitative analysis

Semi-structured interview transcripts were coded by the second author (CvL) using MaxQDA (version 24.7.0) and analysed using thematic analysis with an inductive approach. The process began with open coding, followed by axial coding to group codes into themes and categories, helping to identify patterns within the data. Analysis was conducted in multiple stages to uncover recurring themes across interviews, with regular discussions among the research team to refine the interpretation and structure of codes and themes. To ensure consistency, the first author (RvL) independently coded two transcripts using the final code tree, and any discrepancies were resolved through discussion.

## Supplementary information

Supplementary information

## Data Availability

The data supporting the findings of this study are available from the corresponding author (RvL) upon reasonable request. Due to privacy considerations, the data are not publicly accessible. Metadata are available, and data requests can be submitted via the DataVerseNL repository (10.34894/ITBO7X).

## Code availability

Syntax for the analyses can be found on GitHub (https://github.com/reiniervlinschoten/ai_scribe).
